# The Effect of Infection Precautions on Extended-Spectrum Beta-Lactamase Enterobacteriaceae Colonization Among Nurses in Three Beirut Hospitals

**DOI:** 10.7759/cureus.23849

**Published:** 2022-04-05

**Authors:** Joanna AbiGhosn, Mike AlAsmar, Edmond Abboud, Beth A Bailey, Nicholas Haddad

**Affiliations:** 1 Radiology, Rafik Hariri University Hospital, Beirut, LBN; 2 Department of Internal Medicine, Division of Cardiology, American University of Beirut, Beirut, LBN; 3 Laboratory Medicine, Middle East Institute of Health, Beirut, LBN; 4 Health Services Research, Central Michigan University College of Medicine, Mt. Pleasant, USA; 5 Infectious Disease/Internal Medicine, Central Michigan University College of Medicine, Saginaw, USA; 6 Internal Medicine Residency, Central Michigan University Medical Education Partners, Saginaw, USA

**Keywords:** nursing staff, infection control, hand hygiene, extended-spectrum β-lactamase, contact precaution

## Abstract

Background and objective

Extended-spectrum beta-lactamase-producing Enterobacteriaceae (ESBL-PE) are rapidly emerging worldwide. This study aimed to assess the effect of contact precaution (CP) on ESBL-PE-colonization rates among nurses in three hospitals in Beirut, Lebanon, where ESBL is endemic, in order to define the risk factors for colonization. Accordingly, the ongoing use of CP to prevent ESBL-PE transmission to healthy nurses was evaluated.

Methods

This cross-sectional study was conducted in three hospitals. Hospital 1 required CP, Hospital 2 had recently stopped CP, and Hospital 3 had stopped it three years previously. Questionnaires and stool-collection containers were distributed to all patient care nurses in those three hospitals. The Returned samples were tested using the agar dilution technique.

Results

A total of 269 out of 733 nurses volunteered to participate; 140 met the inclusion criteria (no recent hospitalization, antibiotic use, or known ESBL-PE colonization) and provided samples. Among them, 15% were ESBL-positive. Compared to nurses from Hospital 3, nurses from Hospital 1 were 59% less likely to be colonized, while nurses from Hospital 2 were 62% more likely to be colonized.

Conclusion

In hospitals where CP is in place for ESBL-positive patients, ESBL-PE prevalence in nursing staff was significantly lower. Additionally, a work experience of two to four years increased the odds of ESBL-PE colonization in comparison with longer nursing experience. CP may be a justifiable means of protection against ESBL-PE transmission to healthy nurses. The risk factors for colonization were discontinuation of CP and a shorter clinical work experience.

## Introduction

Extended-spectrum beta-lactamase-producing Enterobacteriaceae (ESBL-PE) are classified as multidrug-resistant organisms (MDROs) by the Centers for Disease Control and Prevention (CDC). These organisms can cause several diseases including urinary tract infections [1], pneumonia [2], bacteremia, and sepsis, which present a challenge to treat due to antimicrobial resistance. This can result in prolonged hospitalizations, an increase in mortality, and high healthcare costs [3-5].

Intestinal colonization has been shown to be the site of carriage of ESBL-PE [6]. The mechanism of person-to-person transmission is via contact with a colonized individual or contaminated hospital environment and via the hands of healthcare workers (HCWs) when hand hygiene is not appropriately performed [7]. Within the bacterial milieu, the resistance is transmitted through genes located on self-transmissible plasmids [8]. 

In a study by Barreto Miranda et al., the median duration of colonization of ESBL-producing Escherichia coli (E. coli) in the human body after exposure is at least six months, and the persistence of colonization depended on multiple factors, such as bacterial genetic factors and patient lifestyle [9]. This cycle of transmission is compounded by the persistence of ESBL producers in the environment of care, particularly if disinfection is not appropriately performed [10].

MDRO-specific CDC infection control guidelines exist for acute-care hospitals and are sometimes also applicable in long-term care settings [11]. These practices fall under two broad categories: administrative, such as compliance with contact precaution (CP), and clinical [11]. However, in environments with high MDRO prevalence in and outside of healthcare settings, the utility of CP is questionable [12]. Despite the recommendations for CP healthcare settings [11,13], more recent studies have challenged its efficacy, and some have even provided data demonstrating a lack of benefit [12,14].

Risk factors predisposing to colonization are well described in the literature, one of the most important being recent antibiotic use (in the preceding 4-12 months) [15,16]. In countries (such as Lebanon) where resources are available, but where regulatory healthcare policy is not enforced, widespread overuse of antimicrobials occurs with consequences fostering microbial resistance [16-18]. Similarly, unregulated utilization of antibiotics in livestock and agriculture may lead to the transmission of resistant strains from farm to fork [19,20]. In addition to antibiotic use, the risk factors for MDROs transmission include previous hospitalization [21], ESBL-PE through household contacts [22], use of antacids [23], diabetes mellitus [21], dialysis, and residence in a long-term care facility [24]. This classical risk of nosocomial ESBL-PE acquisition is compounded by its rapid worldwide emergence in environmental samples unrelated to healthcare [25-27], such as animal cultures [28,29], food sources [30-36], surface water (e.g., in Lebanon after the arrival of refugees) [37,38], and travel to endemic areas [9,39].

Several international studies have evaluated fecal carriage of ESBL-PE from the stools of healthy non-hospitalized individuals [15,40], with one study reporting a prevalence of up to 14% [15]. However, scarce data are available about the prevalence of ESBL-PE in HCWs [41-43]. CP includes the use of gown and gloves before room entry, and removal upon exit, coupled with hand hygiene based on the WHO "Five Moments for Hand Hygiene." The application of CP is a cumbersome practice, with practical and economical disadvantages, as well as adverse psychological effects on the isolated patient [5]. Hence, if it were also found to be ineffective, then eliminating it in healthcare facilities would be comprehensibly justifiable [44]. Moreover, in the new era of severe acute respiratory syndrome coronavirus 2 (SARS-CoV-2) transmission and the worldwide shortage of personal protective equipment (PPE), especially early on in the pandemic, channeling PPE for higher acuity utilization would appropriately reallocate resources.

Most of the rationale behind the use of CP in preventing the transmission of ESBL-producers is deduced from the literature pertaining to methicillin-resistant Staphylococcus aureus [45]. The efficacy of contact isolation in the prevention of ESBL transmission in outbreak settings has been proven [46-48]. However, a similar efficacy in settings with a high risk of HCW colonization with ESBL has not been evaluated.

The primary objective of this study was to determine if CP is associated with reduced rates of ESBL-PE colonization in nursing staff. The secondary objective was to identify additional risk factors for ESBL-PE colonization among nursing staff.

## Materials and methods

Participants

The study participants were nurses recruited between July and November 2017 from three hospitals in Beirut, who were selected based on strategic geographic location and catchment areas, and also due to some of the authors having clinical privileges in them. Hospital 1 required contact isolation for ESBL-carriers, Hospital 2 had stopped this practice a month earlier (as per the decision of their infection control committee), and Hospital 3 had stopped isolation for ESBL carriers three years prior to the study period. Of note, in Hospital 2, after stopping CP for ESBL-carriers, there was no change in any other infection control practices otherwise, particularly in cleaning and disinfection procedures. The three hospitals were otherwise comparable with respect to the number of beds (between 100 and 200), patient population and profiles, and the number of employees; besides, all three of them were community-based teaching hospitals (not university hospitals).

All nurses on staff at the three participating hospitals were offered participation. Exclusion criteria were hospitalization for more than two days within the past 12 months, known colonization with ESBL-PE or known contact with ESBL-positive household members, and any utilization of an antimicrobial agent of any class within the preceding four months.

Procedures and measures

Prior to the initiation of enrolment and data collection, the study was presented to the nursing leadership in each of the three hospitals to encourage and maximize nursing staff participation. Ethical conduct of research was strictly followed in every step, which involved explaining the study objectively, sharing the methodology clearly via written material, promoting it on each floor via flyers written in clear, understandable instructions, and ensuring the preservation of result confidentiality. A numbered sterile cup for a stool sample and a questionnaire with the same number were placed together in a plastic bag and distributed to all nursing staff, through the nursing supervisor of each nursing unit. Of note, the hand hygiene rates of nursing staff in the three hospitals had consistently been above 85%, as documented in the infection control records based on observations.

The questionnaires collected the following information: age and gender, length of time working as a clinical nurse, clinical work setting over the preceding six months, hospitalizations within the preceding 12 months, antibiotic treatment within the preceding four months, personal colonization status by ESBL-PE if known, and contact with household members whose colonization status was known to be positive for ESBL-PE.

The questionnaire was completed at home by the volunteering participants and returned with the stool specimen. Fresh stool samples were returned to the lab of each hospital where they were stored at 4 °C. The samples were retrieved within 24 hours, and the questionnaire was reviewed. If the participants fulfilled the inclusion criteria based on the questionnaire, the samples were transported to a single laboratory for processing to ensure standardized results.

Stool samples were inoculated on a MacConkey agar plate and incubated at 37 °C for 24 hours. Lactose fermenter colonies (E. coli, Klebsiella spp., Citrobacter spp., Enterobacter spp.) were picked and suspended in a sterile normal saline solution to make a 0.5 Macfarland bacterial suspension. Using a cotton swab, the bacterial suspension was evenly and uniformly streaked on a Mueller-Hinton agar plate. A ceftazidime disk (30 mcg), a cefotaxime disk (30 mcg), and a cefepime disk (30 mcg) were placed around an amoxicillin-clavulanic acid disk (20-10 mcg) located in the center of the plate and then incubated at 37 °C for 24 hours. The presence of a bacterial inhibition area between the oxyimino cephalosporin disks and the amoxicillin-clavulanic acid disk was considered a positive test result. The cause of this inhibition area is the synergism between the oxyimino cephalosporins and the clavulanic acid in deactivating the ESBL secreted by the bacteria. Bacteria that were resistant to oxyimino cephalosporins (according to the disk diffusion method, CLSI Document M100-S19) and showed synergism with clavulanic acid were identified as ESBL-positive bacteria.

Approval and oversight

Administrative support and informed consent from the leadership in each hospital were secured after the full disclosure of study details, and by assuring to maintain the anonymity of the hospital and staff members along the course of the study and after the results were analyzed. Individual verbal consent was also obtained from each participating nurse, who had the opportunity to ask any question about the study. All questions were answered by the primary investigator.

Results from nurses’ stool cultures were kept confidential. They were made available solely to each individual nurse by e-mail or phone messages and were not shared otherwise, namely with other hospital staff, administration, or the hospital’s infection control departments. Information as to what the result meant was provided upon request to each of the recipients of those results.

Data analysis

Based on Bassyouni et al. [41], we fixed our expected frequency of prevalence of ESBL-PE colonization at 21% and our precision level at ±5%; based on this, we obtained a minimum sample size of 255 for a confidence level of 95% to detect rate differences between the three hospitals.

All statistical analyses were carried out using the SPSS software version 21 (IBM, Armonk, NY). We performed descriptive statistics by reporting means (± standard deviations) for continuous variables and frequencies (percentages) for categorical ones. All factors potentially associated with ESBL-PE colonization, including hospital type, were first analyzed using bivariate statistics (Student’s t-test for continuous variables and Pearson’s chi-square or Fisher’s exact test for categorical variables). To assess the independent effect of hospital type, we performed binary logistic regression using a generalized estimating equation model to account for the correlation between measures taken from the same hospital with the culture result (ESBL-PE colonization) as the dependent variable and potential associated factors as covariates. We chose as covariates the factors associated with the dependent variable with a p-value <0.2 in the bivariate analysis. Adjusted odds ratios (OR) and 95% confidence intervals (CI) were reported. For all analyses, a p-value <0.05 was considered statistically significant.

## Results

**Figure 1:**
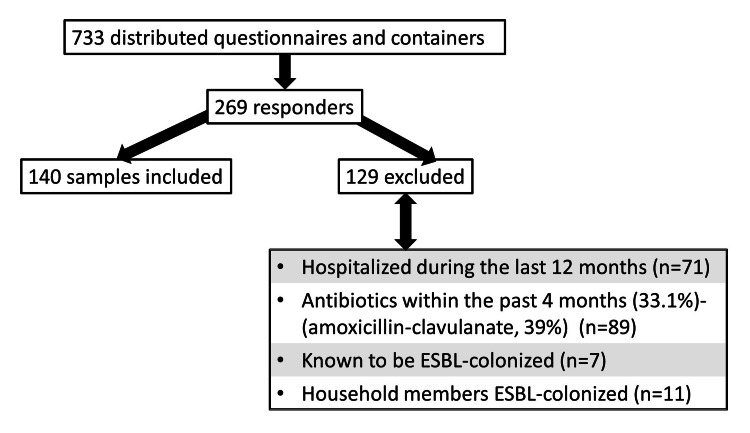
Flow diagram of the study inclusion and exclusion criteria The numbers for the excluded do not sum up to 129 as the exclusion criteria were not mutually exclusive ESBL: extended-spectrum beta-lactamase

All 733 of the nurses at the three hospitals received questionnaires and containers, and a total of 269 nurses (36.7%) returned the questionnaire with the samples. Of the 269 samples, 129 (48%) were excluded based on exclusion criteria, as detailed in Figure 1. Thus, the final analysis was based on 140 completed questionnaires and stool sample data.

**Figure 2:**
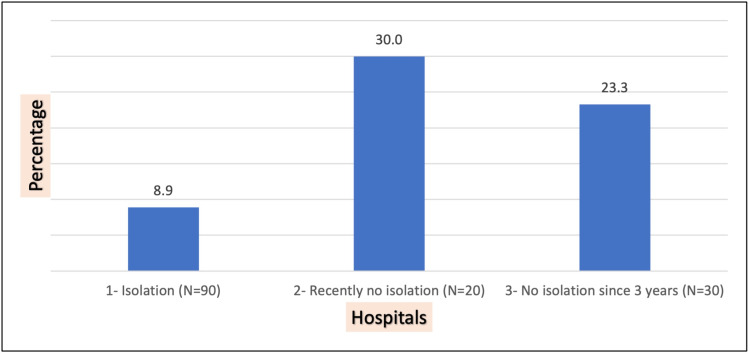
Proportion of ESBL-positive cultures in each of the three study hospitals N=140 Of the 140 cultures performed, 21 (15%) were positive. As shown in Figure 2, nurses working in Hospital 1 (isolation being followed) were significantly less colonized (p=0.016) than those working in Hospital 2 (recently stopped isolation) and Hospital 3 (no isolation for the prior three years). The significance noted herein could be due to a number of possible confounding factors such as the nurses' living conditions outside of the hospital as well as their food and water supply, eating habits, socioeconomic status, etc. Yet, it was a significant finding in our small study, which could be clarified in larger studies in the future ESBL: extended-spectrum beta-lactamase

Participant characteristics are detailed in Table 1 and Figure 2. Approximately half of the participants were working in Hospital 1 (i.e., where contact isolation was practiced), more than two-thirds were female, and the majority of respondents had more than six years of nursing experience.

**Table 1:** Sample characteristics per hospital P-values are derived from t-tests for continuous variables, and chi-square tests for categorical variables SD: standard deviation

Characteristics	Hospital 1 (n=136)	Hospital 2 (n=49)	Hospital 3 (n=77)	P-value
Age, mean ± SD	37.9 ± 9.1	32.8 ± 7.5	33.3 ± 9.4	<0.001
Females, n (%)	94 (66.7)	36 (73.5)	56 (70.9)	0.623
Seniority level, n (%)				
<2 years	22 (15.7)	5 (10.2)	12 (15.2)	0.674
2–4 years	10 (7.1)	5 (10.2)	11 (13.9)	
4–6 years	10 (7.1)	5 (10.2)	5 (6.3)	
>6 years	98 (70.0)	34 (69.4)	51 (64.6)	

Bivariate analyses for other factors associated with ESBL colonization are shown in Table 2. Nurses who were ESBL-colonized were significantly younger than nurses without ESBL-colonization. Nurses with less than four years of clinical experience tended to be more colonized than other nurses but this difference did not reach statistical significance.

**Table 2:** Characteristics of nurses by culture result N=140 P-values are derived from t-tests for continuous variables, and chi-square tests for categorical variables ESBL-PE: extended-spectrum beta-lactamase-producing Enterobacteriaceae; SD: standard deviation

Characteristics	Nurses not colonized with ESBL-PE (n=119)	Nurses colonized with ESBL-PE (n=21)	P-value
Age (years)			
mean ± SD	36.6 ± 8.4	31.0 ± 8.2	0.006
Gender, n (%)			
Female	82 (86.3)	13 (13.7)	0.526
Male	37 (82.2)	8 (17.8)	
Seniority level, n (%)			0.075
<2 years	14 (73.7)	5 (26.3)	
2–4 years	8 (66.7)	4 (33.3)	
4–6 years	10 (90.9)	1 (9.1)	
>6 years	87 (88.8)	11 (11.2)	
Clinical setting in the last 6 months, n (%)			0.24
Multiple floors	24 (92.3)	2 (7.7)	
Medicine	26 (74.3)	9 (25.7)	
Surgery	28 (87.5)	4 (12.5)	
Critical care	25 (89.3)	3 (10.7)	
Psychiatry	1 (50.0)	1 (50.0)	
Interventional	12 (85.7)	2 (14.3)	

Finally, the multivariable analysis showed a significant association between hospital type and ESBL-PE colonization after controlling for both age and seniority level, as detailed in Table 3. Compared to nurses working in a hospital with no isolation procedures for the last three years, nurses working in a hospital with isolation required were 59% less likely to have a positive culture. Additionally, compared to nurses working in a hospital with no isolation precautions for the last three years, nurses working in a hospital that had recently discontinued isolation for ESBL-PE carriers were 62% more likely to have a positive culture. Finally, seniority remained significant in the model, with nurses with two to four years of experience being 2.6 times more likely to have a positive culture than nurses who have worked for more than six years.

**Table 3:** Multivariable analysis (GEE) of factors predicting ESBL-PE colonization N=140 ESBL-PE: extended-spectrum beta-lactamase-producing Enterobacteriaceae; OR: odds ratio

Factors	Adjusted OR	95% confidence interval		P-value
		Lower bound	Upper bound	
Age	0.941	0.856	1.034	0.207
Hospital				
1 - Isolation	0.41	0.343	0.49	<0.001
2 - Recently no isolation	1.623	1.377	1.914	<0.001
3 - No isolation for the previous 3 years	Reference			
Seniority level				
<2 years	1.578	0.768	3.241	0.214
2–4 years	2.599	1.179	5.731	0.018
4–6 years	0.597	0.147	2.416	0.469
>6 years	Reference			

## Discussion

This is the first study conducted in Lebanon to examine the prevalence and risk factors for ESBL-PE colonization in nursing staff in relation to different hospital infection control practices with ESBL-colonized and ESBL-infected patients.

Hospital 1, where the CP policy was still being followed, had the lowest prevalence of colonization among nursing staff. We believe that it was likely due to a favorable effect of CP. Surprisingly, Hospital 2, where CP was removed a month prior to the sample collection, had the highest prevalence of ESBL-PE colonization. This observation, as important as it is in this small study, has a lot of possible confounding factors as mentioned above. Having found no explanation for this phenomenon in the literature, we propose a few possible reasons for this highest prevalence.

Firstly, we theorize that this may be explainable by what we describe as a "flooding" effect, whereby the gastrointestinal tracts of nursing staff were rather quickly populated by resistant organisms "new" to their systems after the lifting of CP. Second, it is possible that nursing staff may have felt more "secure" that isolation was no longer required, and consequently became less compliant with strict adherence to standard precautions. A third possible explanation is that environmental contamination may have played a role [49-51], as ESBL-PE can survive on surfaces for many months if disinfection is not well performed [10,52], leading to concerns about direct causality. Consequently, evaluating the environmental contamination and monitoring the established cleaning processes are critical [53]. Hence, upon discontinuation of CP for ESBL-PE colonized/infected patients in any healthcare organization, it is recommended to provide enhanced and ongoing education to staff about the need for compliance with established disinfection procedures, coupled with monitoring of compliance [48], in a concerted effort to mitigate the potential for increased MDRO transmission. With regard to finding the source of ESBL-PE in the guts of nursing staff, molecular typing may have a role [48], although this was not performed in our study as it was not part of the original study design.

A study conducted by Bassyouni et al. in Egypt in 2013, published in 2015, demonstrated that the rate of fecal carriage of ESBL-PE in HCWs practicing standard precautions was 21% [41], which is comparable to what we found despite the smaller number of samples in our study.

In this study, the highest rates of ESBL-PE colonization were in participants with two to four years of experience in clinical work as nurses, especially compared to those who have worked longer than four years. Our interpretation of this observation is that less experienced nurses may get colonized upon entering the hospital environment, especially in settings where contact isolation is not performed. The ESBL-PE prevalence data for nurses in hospitals that do not require CP in our study are comparable to rates of ESBL-positive hospital samples as published by the Lebanese Society of Infectious Diseases and Clinical Microbiology in 2016. This was a retrospective study of 16 different Lebanese hospitals between January 2011 and December 2013 [54], where the ESBL-PE rate of E. coli isolates was 32.3% and that of Klebsiella was 29.2%. The comparable prevalence between clinical samples from this study, and the prevalence among nursing staff of hospitals without CP would indirectly support the idea that hospital CP practices impart at least some protection against MDRO colonization. We find a similarity between the prevalence data of the hospital still requiring CP to that of a Lebanese nursing home data [55], which is lower than the hospital resistance data described above [54]. This is likely due to the nursing home prevalence being more reflective of community prevalence data as opposed to acute-care hospital data.

It is not surprising to see a high rate of colonization of ESBL producers in the Lebanese community, where those nurses live. This is where the dissection of the sources of ESBL, whether from the community or nosocomial acquisition, becomes a challenging issue that cannot be answered microbiologically or epidemiologically at this point and with the currently available resources in the country. Of note, the overall prevalence of ESBL in Enterobacteriaceae in microbiologic data from each of those hospitals was around 40% at the time of conducting this study.

Interestingly, a study by Challita et al. in 2015 demonstrated an ESBL E. coli prevalence of 7.7% in a Lebanese nursing home [55]. We are not able to explain this lower-than-average prevalence data point, especially in a cohort of institutionalized elderly patients. However, we do find a reflection of this prevalence in our nursing staff cohort at Hospital 1, where CP continues to be practiced. Hence, we question whether the effect of healthcare acquisition is mitigated by strict adherence to CP, ultimately resulting in a lower prevalence of ESBL colonization among nurses, with 8.9% ESBL positive carriers in Hospital 1, comparable to the 7.7% ESBL E. coli in the Challita study [55].

This study has several limitations. Primarily, the level of nursing staff participation was somewhat low (269 volunteered from a potential pool of 733 nurses) and participation was not congruous between the three hospitals. This could have introduced bias as those who did not respond are very likely to differ in characteristics of interest from those who responded. Our sample contained significantly more nurses from Hospital 1 (52.4% of our sample) compared to Hospital 2 (18.2%) and Hospital 3 (29.4%). We theorize that this higher rate of participation of Hospital 1 nursing staff may have been fueled by an inherent interest to confirm that the laborious practices of donning gowns and gloves for each ESBL-PE patient encounter were indeed leading to a transmission-protective effect both for them and for the patients. Additionally, another unstated reason could be the ultimate possibility of discontinuing this practice if it were found to be ineffective. Finally, the overall low rate of participation could be due to the unappealing call to provide stool samples. Despite the potential limitation of lower participation rates and unequal sample sizes, we were adequately powered, and our group differences were significant, revealing the importance of CP as a protective way of curtailing transmission of ESBL-PE from patients to nursing staff. Another limitation is that other infection control practices such as disinfecting procedures, number of sinks for handwashing, antimicrobial use restrictions, and maintaining adequate staffing levels were not compared between the three hospitals.

Although we believe our exclusion criteria played a significant role in the elimination of potential confounders, they were rather strict; 33.1% of volunteers were excluded due to the recent intake of an antimicrobial agent within the preceding four months. Had those nurses been included, we might have been able to derive some useful information from them. Of note, among the excluded staff due to recent antimicrobial use, the most common agents were amoxicillin-clavulanic acid (39%), cephalosporins (10%), and fluoroquinolones (10%). In a country like Lebanon, where antibiotics are routinely purchased without a prescription, such a high rate of antibiotic use may not be surprising although it is disturbing, particularly in a working population of healthcare professionals. We suggest that the overly high use of amoxicillin-clavulanic acid is likely due to its low price, easy availability, and familiarity with its use [56].

Lastly, we did not utilize PCR technology to analyze specific ESBL gene types in any hospital [57]. Such a tool would have shed further light on the specific ESBL enzymes in studies like ours, and that in turn may have explained certain epidemiologic trends (nosocomial vs. community sources, care site-specificity, environmental acquisition, etc.).

Despite these limitations, we do believe this study provides an important confirmatory observation regarding the protective impact that CP has against the transmission of ESBL-PE strains among the nursing staff.

## Conclusions

Based on our findings, CP does lead to a positive impact on reducing the transmission risk of ESBL-producing Gram-negatives to nursing staff. The risk factors for colonization were the removal of CP (including recent discontinuation) and a shorter clinical work experience, i.e., two to four years. Further studies are required to define the role of hospital environmental contamination vs. community contribution as well as the role of ESBL-PE clonality.
